# Evaluation of methods for predicting the topology of β-barrel outer membrane proteins and a consensus prediction method

**DOI:** 10.1186/1471-2105-6-7

**Published:** 2005-01-12

**Authors:** Pantelis G Bagos, Theodore D Liakopoulos, Stavros J Hamodrakas

**Affiliations:** 1 Department of Cell Biology and Biophysics, Faculty of Biology, University of Athens, Panepistimiopolis, Athens 15701, Greece

## Abstract

**Background:**

Prediction of the transmembrane strands and topology of β-barrel outer membrane proteins is of interest in current bioinformatics research. Several methods have been applied so far for this task, utilizing different algorithmic techniques and a number of freely available predictors exist. The methods can be grossly divided to those based on Hidden Markov Models (HMMs), on Neural Networks (NNs) and on Support Vector Machines (SVMs). In this work, we compare the different available methods for topology prediction of β-barrel outer membrane proteins. We evaluate their performance on a non-redundant dataset of 20 β-barrel outer membrane proteins of gram-negative bacteria, with structures known at atomic resolution. Also, we describe, for the first time, an effective way to combine the individual predictors, at will, to a single consensus prediction method.

**Results:**

We assess the statistical significance of the performance of each prediction scheme and conclude that Hidden Markov Model based methods, HMM-B2TMR, ProfTMB and PRED-TMBB, are currently the best predictors, according to either the per-residue accuracy, the segments overlap measure (SOV) or the total number of proteins with correctly predicted topologies in the test set. Furthermore, we show that the available predictors perform better when only transmembrane β-barrel domains are used for prediction, rather than the precursor full-length sequences, even though the HMM-based predictors are not influenced significantly. The consensus prediction method performs significantly better than each individual available predictor, since it increases the accuracy up to 4% regarding SOV and up to 15% in correctly predicted topologies.

**Conclusions:**

The consensus prediction method described in this work, optimizes the predicted topology with a dynamic programming algorithm and is implemented in a web-based application freely available to non-commercial users at .

## Background

Transmembrane proteins are divided to date into two structural classes, the α-helical membrane proteins and the β-barrel membrane proteins. Proteins of the α-helical membrane class have their membrane spanning regions formed by hydrophobic helices which consist of 15–35 residues [1]. These are the typical membrane proteins, found in cell membranes of eukaryotic cells and bacterial inner membranes [1]. On the other hand, β-barrel membrane proteins, have their transmembrane segments, formed by antiparallel β-strands, spanning the membrane in the form of a β-barrel [2,3]. These proteins are found solely in the outer membrane of the gram-negative bacteria, and presumably in the outer membranes of mitochondria and chloroplasts, a fact, perhaps, explained by the endosymbiotic theory [4-7]. Transmembrane protein topology prediction has been pursued for many years in bioinformatics, mostly focusing on the α-helical membrane proteins. One reason for that, is that α-helical transmembrane segments are more easily predicted by computational methods, due to the easily detectable pattern of highly hydrophobic consecutive residues, and the application of simple rules as the "positive-inside rule" [8]. On the other hand, another reason is the relative abundance of α-helical membrane proteins compared to that of the β-barrel membrane proteins. This discrepancy, is present in both the total number of membrane proteins in complete genomes, an also in the datasets of experimentally solved 3-dimensional structures. Currently, the number of structures of outer membrane proteins known at atomic resolution raises rapidly, due to improvements in the cloning and crystallization techniques [9]. This, fortunately, gave rise to an increase of the number of prediction methods and the online available web-predictors. The first computational methods that were deployed for the prediction of the transmembrane strands were based on hydrophobicity analyses, using sliding windows along the sequence, in order to capture the alternating patterns of hydrophobic-hydrophilic residues of the transmembrane strands [10,11]. Other approaches included the construction of special empirical rules using amino-acid propensities and prior knowledge of the structural nature of the proteins [12,13], and the development of Neural Network-based predictors to predict the location of the Cα's with respect to the membrane [14]. The major disadvantages of these older methods, were the limited training sets that they were based on, and the reduced capability to capture the structural features of the bacterial outer membrane proteins, especially when it comes to sequences not having similarity with the proteins of the training set. During the last few years, other more refined methods, using larger datasets for training, appeared. These methods, include refined Neural Networks (NNs), [15,16], Hidden Markov Models (HMMs) [17-21] and Support Vector Machines (SVMs) predictors [22]. Some of these methods are based solely on the amino acid sequence and others use also as input evolutionary information derived from multiple alignments. Other popular methods such as the method of Wimley [23] and BOMP [24] do not explicitly report the transmembrane strands, but instead they are oriented towards genome scale discrimination of β-barrel membrane proteins.

In this work, we evaluate the performance of the available prediction methods to date. Using a non-redundant dataset of 20 outer membrane β-barrel proteins, with structures known at atomic resolution, we compare each predictor in terms of the per-residue accuracy (using the correctly predicted residues, and the Mathews correlation coefficient [25]) and that of the strands' prediction accuracy measured by the segments overlap measure (SOV) [26]. We also report the number of the correctly predicted topologies (i.e. when both strands localization and orientation of the loops are correctly predicted). We conclude, that the recently developed Hidden Markov Model methods HMM-B2TMR [17], ProfTMB [21] and PRED-TMBB [20], perform significantly better than the other available methods. We also conclude that the prediction accuracy is affected significantly, if the full sequences (including long N-terminal and C-terminal tails and the signal peptide) are used for input and not only the transmembrane β-barrel domain. This finding is again more profound when referring to the NN and SVM predictors, since the regular grammar of the HMMs maps successfully the model topology to the proteins' modular nature. Finally, we developed a consensus prediction method, using as input the individual predictions of each algorithm, and we conclusively show that this approach performs better, in all the measures of accuracy, compared to each individual prediction method separately. Although consensus methods have proven to be more accurate in the past, in the case of α-helical membrane proteins [27-29] and also for secondary structure prediction of globular, water soluble proteins [30-32], this is the first time that such a method is applied to β-barrel outer membrane proteins.

## Results and discussion

The results obtained from each individual algorithm, on the test set of the 20 proteins are summarized in Table 1. It is obvious that all of the methods perform worse for the measures of per-segment accuracy in the case of full-length sequences. On the other hand, for measures of per-residue accuracy, most of the methods perform better in the case of full-length sequences, a fact already mentioned in [21]. This is explained, considering the fact that when using full-length sequences, more non-transmembrane residues are predicted correctly, thus increasing the fraction of correctly predicted residues and the correlation coefficient. Furthermore, when ranking the different methods PRED-TMBBposterior performs better, followed by HMM-B2TMR and ProfTMB. PRED-TMBBnbest, performs slightly worse than PRED-TMBBposterior in terms of per-residue accuracy and SOV, but is inferior to the other top-scoring HMMs in terms of the correctly predicted topologies. In order to assess the statistical significance of these observations and draw further safe conclusions, we should rely on a statistical analysis of the results obtained.

The MANOVA test (Table 2A) yields a highly significant p-value for both the 2 independent variables (p < 10^-4^). This means, that there is truly a difference in the vector of the five measured attributes across the different methods and the type of sequence that we use as input. By including in the model the interaction term between the two factors, we get a marginally insignificant p-value (p = 0.0619), indicating that some of the methods behave differently with input sequences of different type. Examining each one of the attributes independently (Table 3A), we observe that the type of the input sequence does not influence significantly the effect on all the measures of per-residue accuracy (correctly predicted residues and the correlation coefficient, p-values equal to 0.9444 and 0.0224 respectively) but, instead, influences a lot the per-segment measures such as SOV (p < 10^-4^), correctly predicted topologies (p = 0.0193) and correct barrel size (p = 0.0001). In all cases, the type of the method is a highly significant factor (p < 10^-4^), reflecting the fact that there are true differences in the performance of the methods. The interaction term in the ANOVA is significant only for the SOV measure (p = 0.0272), and marginally significant for the correctly predicted residues (p = 0.402). However, these results do not provide us with a clue as to which method performs better (or worse) than the others; it states that one or more methods depart significantly from the mean. The ranking of the methods has to be concluded by observing Table 1.

In order to discover the statistically significant differences between the methods, we proceeded by grouping the methods according to the type of the algorithm they utilize. This way, we grouped together the HMM-based methods (HMM-B2TMR, PRED-TMBB, ProfTMB and BETA-TM) and the NN and SVM-based methods (TMBETA-NET, B2TMPRED, PSI-PRED and TBBPred). Thus, instead of having a factor with 8 levels describing the methods, we now have a factor with 2 levels (HMM and not HMM). The MANOVA test (Table 2B) once again yields a statistically significant result, for both the 2 factors (p < 10^-4^) and the interaction term (p = 0.0025), giving us a clear indication that the visually observed superiority of the HMM-based methods has a statistically significant justification. The statistically significant interaction of the 2 factors, furthermore suggests that the decrease in some of the measured attributes when submitting full-length sequences, is smaller (if anything) for HMM-based methods than for the NN and SVM-based ones. In fact, considering the three top-scoring HMM methods, we observe that the per-segment measures are not influenced from the type of the input sequence whereas the per-residue measures are significantly increased with full-length sequences as input, reflecting the fact that more non-transmembrane residues are correctly predicted, as noticed already in [21]. Considering each one of the measures of accuracy with ANOVA (Table 3B), the type of the method is a highly significant factor in all of the tests, and the type of the input sequence highly significant for the per-segment measures of accuracy. The interaction term is highly significant for SOV (p = 0.0011) and marginally insignificant for correctly predicted residues (p = 0.052).

These findings suggest, that the HMM-based predictors perform better, on average, than the NN and SVM-based methods, in almost all of the measured attributes. We should mention here, that the difference between HMM and NN/SVM methods is larger for the measures of per-segment accuracy than for per-residue accuracy. Even the simplest and less accurate HMM-based method, BETA-TM, that uses single sequence information compares favorably to the refined NN/SVM methods that use profiles derived from multiple alignments. As a matter of fact, only B2TMPRED, which uses a dynamic programming algorithm to refine the prediction, predicts more accurately than BETA-TM the correct topology and/or the barrel size of the proteins, but still cannot reach the accuracy of the other HMM-based methods. Furthermore, the HMM-based methods are not influenced significantly whether full-length sequences or just the β-barrel domains are submitted for prediction. Interestingly, the NN/SVM methods, often falsely predict the signal peptide sequences as transmembrane strands in the precursors whereas HMMs do not. This observation is consistent with the theory regarding the nature of HMM and NN-based methods. Thus, it is consistent with the fact that the regular grammar of the HMMs can capture more effectively the temporal variability of the protein sequence and map successfully the proteins' modular nature to a mathematical sound model. Therefore, it is not surprising that also for α-helical membrane proteins' topology prediction the best available predictors are those based on HMMs [33]. On the other hand, NN methods are more capable of capturing long-range correlations along the sequence. This results to the correct identification of an isolated strand, but since the β-barrel proteins follow strict structural rules, the modular nature of the barrels is captured more effectively by HMMs. NNs may often falsely predict isolated transmembrane strands in non-barrel domains or predict strands with a non-plausible number of residues or even barrels with an odd number of strands. From a structural perspective, it is also of great interest to consider that the repetitive structural domains of β-barrels are the β-hairpins whereas the α-helical membrane proteins counterparts are the isolated hydrophobic helices often connected by loop regions of arbitrary length.

These observations, will have a significant impact not only on isolated predictions for one or few proteins, but also on predictions for sequences arising from genome projects where one expects to have the precursor sequences. Thus, predictions on such sequences will be more reliable, when obtained from HMM-predictors rather than NN and SVM-based ones. However, the performance of even the best currently available predictors are not as good as the predictions obtained for α-helical membrane proteins [33]. This is somewhat expected, and has a simple interpretation considering the grammatical structure of the short amphipathic transmembrane β-strands as opposed to the longer and highly hydrophobic transmembrane α-helices [1].

One issue that was not possible to investigate statistically is that of the use of evolutionary information in the form of profiles derived from alignments. It is well known, that the inclusion of information arising from alignments, increases significantly the performance of secondary structure prediction algorithms [34]. This was exploited in the past, in the case of α-helical membrane protein prediction [35,36], and it was investigated thoroughly in a recent work [37]. However, for β-barrel membrane proteins there is not such a clear answer. The authors of the methods that use evolutionary information [15,17,21] justified their choice showing that the inclusion of alignments as input, improves the performance of their models up to 18%. Furthermore, we showed here that NN-based methods, using multiple alignments (B2TMPRED) perform significantly better, compared to similar methods that are relying on single sequences (TMBETA-NET). However, the top scoring HMM method, PRED-TMBB, performs comparably to the other HMM methods that are using evolutionary information, even though it relies on single sequence information. This finding may be explained considering the choice of the training scheme for PRED-TMBB, since it is the only method trained according to the CML criterion, and with manually curated annotations for the transmembrane strands. However, it raises an important question as to whether the prediction accuracy, could be improved more by using evolutionary information, or not. Future studies on this area will reveal if improvements in the prediction could arise by combining evolutionary information with appropriate choice of training schemes, or if we have eventually reached a limit of the predictive ability for β-barrels membrane proteins, and we depend only on the advent of more three-dimensional representative structures.

Comparing the performance of individual methods, one has to keep in mind several important aspects of the comparison. From the one hand, the limited number of β-barrel membrane proteins known at atomic resolution, resulted in having a test set, that includes some (or all) of the proteins used for training each individual method or a close homologue. This does not imply that the comparison of the methods is biased (regarding the ranking), but that the absolute values of the measures of accuracy may be influenced. Thus, when it comes to newly solved structures, we may expect somewhat lower rates in the measures of accuracy for all methods examined. On the other hand, when comparing the results of the individual methods, as they appear in the original publications, we observe some discrepancies. These arise, mainly due to the fact, that when reporting results of a prediction method, the authors usually report the measures of accuracy obtained in the jackknife test (leave one out cross-validation test). Furthermore, the authors of the individual methods report the measures of accuracy obtained using as input different types of sequences, and comparing using as observed different annotations for the transmembrane strands. For instance, other authors report measures of accuracy obtained from the β-barrel domain of the proteins, others from the sequences deposited in PDB, and others report also the results from precursor sequences. As for the observed transmembrane strands used for comparisons, most of the authors used the annotations for the strands found in PDB, and only PRED-TMBB used manually annotated segments that resemble better the part of the strand inserted into the lipid bilayer. The last observation, partly explains the better prediction accuracy obtained by PRED-TMBB, mainly in the measures of per-residue accuracy (correctly predicted residues and correlation coefficient).

One important result of this study is the development of the consensus prediction method, for predicting the transmembrane strands of β-barrel membrane proteins. Even though consensus prediction has been proved to be a valuable strategy for improving the prediction of α-helical membrane proteins [27,29,38], no such effort has been conducted before, for the case of transmembrane β-barrels. A consensus of all of the available methods, does not improve the prediction accuracy compared to the top-scoring methods, indicating that there is a considerable amount of noise in the individual predictions, originating mainly from the low-scoring methods. However, when using the three top-scoring HMM methods (PRED-TMBB, HMM-B2TMR and ProfTMB) along with one or more of the best performing NN/SVM methods (B2TMPRED, TBBPred-SVM, TBBPred-NN and TBBPred-Combined) we get impressive results, outperforming the top-scoring methods in almost all measured attributes. As it is obvious from Tables 1 and 4, the consensus prediction method performs better than each one of the individual predictors. The improvement ranges from a slight improvement around 1% for the correctly predicted residues and correlation coefficient, up to 4% for SOV and 15% for the correctly predicted topologies. We should note that these particular results were achieved using PRED-TMBBposterior, ProfTMB, HMMB2TMR, B2TMPRED and TBBPred-NN, but other combinations of the aforementioned methods perform similarly (Table 4). This large improvement in the measures of per-segment accuracy is an important finding of this study.

However, in the web-based implementation of the consensus prediction method, we allow the user to choose at will the methods that will be used for the final prediction. This was decided for several reasons: Firstly, for a newly found protein, we might have larger variations on the predictions, and we could not be sure if the choice of different algorithms will give better results or not. Secondly, the different predictors are not sharing the same functionality and availability. For instance, some predictors respond by e-mail (B2TMPRED, PSIPRED), most of the others by http (PRED-TMBB, BETA-TM, TMBETA-NET etc), and others may be downloaded and run locally (ProfTMB, PSIPRED), whereas one of the top-scoring methods (HMM-B2TMR) is available as a commercial demo only, requiring a registration procedure. These facts, forced us not to have a fully automated server (but instead we require the user to cut 'n paste the predictions) but also to allow flexibility on the chosen methods, and let the user decide alone which methods he will use. For this reason, we also give to the users the opportunity to provide, if they wish, custom predictions. This way, a user may choose to use another method, that will come up in the future, or, alternatively, to use manually edited predictions.

## Conclusions

We have evaluated the currently available methods, for predicting the topology of β-barrel outer membrane proteins, using a non-redundant dataset of 20 proteins with structures known at atomic resolution. By using multivariate and univariate analysis of variance, we conclude that the HMM-based methods HMM-B2TMR, ProfTMB and PRED-TMBB perform significantly better than the other (mostly NN-based) methods, in both terms of per-residue and per-segment measures of accuracy. We also found, a significant decrease in the performance of the methods when full-length sequences are submitted for prediction, instead of just the β-barrel domain. However, the HMM-based methods are more robust as they were found largely unaffected by the type of the input sequence. This is an important finding that has to be taken in account, not only in the cases of single proteins' predictions, but mostly in cases of predictions performed on precursor sequences arising from genome projects. Finally, we have combined the individual predictors, in a consensus prediction method, that performs significantly better even than the top-scoring individual predictor. A consensus prediction method is for the first time been applied for the prediction of the transmembrane strands, of β-barrel outer membrane proteins. The consensus method, is freely available for non-commercial users at , where the user may choose which of the individual predictors will include, in order to obtain the final prediction.

## Methods

### Data sets

The test set that we used has been compiled mainly with consideration of the SCOP database classification [39]. In particular, all PDB codes from SCOP that belong to the fold "Transmembrane beta-barrels" were selected, and the corresponding structures from the Protein Data Bank (PDB) [40] were obtained. For variants of the same protein, only one solved structure was kept, and multiple chains were removed. The structure of the β-barrel domain of the autotransporter NalP of *N. meningitidis *[41] was also included, which is not present in the SCOP classification although it is clearly a β-barrel membrane protein. The sequences have been submitted to a redundancy check, removing chains with a sequence identity above a certain threshold. Two sequences were considered as being similar, if they demonstrated an identity above 70% in a pairwise alignment, in a length longer than 80 residues. For the pairwise local alignment BlastP [42] was used with default parameters, and similar sequences were removed implementing Algorithm 2 from [43]. The remaining 20 outer membrane proteins constitute our test set (Table 5).

The structures of TolC [44], and alpha-hemolysin [45], were not included in the training set. TolC forms a trimeric β-barrel, where each monomer contributes 4 β-strands to the 12-strand barrel. Alpha-hemolysin of *S. aureus *is active as a transmembrane heptamer, where the transmembrane domain is a 14-strand antiparallel β-barrel, in which two strands are contributed by each monomer. Both structures are not included in the fold "transmembrane beta-barrels" of the SCOP database. In summary, the test set (Table 5), includes proteins functioning as monomers, dimers or trimers, with a number of transmembrane β-strands ranging from 8 to 22, and is representative of the known functions of outer membrane proteins to date.

In order to investigate the effect of the full sequence on the different predictors, we conducted two sets of measurements. In the first place, all proteins were submitted to the predictors, in their full length. We chose not to remove the signal peptides, considering the fact that completely unannotated sequences, mostly originating from genome projects, are most likely to be submitted to predictive algorithms, in their pre-mature form. Of the 20 sequences constituting our set, 4 belonging to the family of TonB-dependent receptors, namely FhuA [46], FepA [47], FecA [48] and BtuB [49] posses a long (150–250 residues) N-terminal domain that acts as a plug, closing the large pore of the barrel. This domain is present in all four of the structures deposited in PDB. One of the proteins of our dataset, OmpA possesses a long 158 residue C-terminal domain falling in the periplasmic space, which is absent from the crystallographically solved structure [50]. Finally, the Secreted NalP protein, possesses a very long, 815 residues in length, N-terminal domain that is being transported to the extracellular space passing through the pore formed by the autotransporter β-barrel pore-forming domain, of which we have the crystallographically solved structure [41]. For the second set of measurements, for all proteins constituting our dataset we extracted only the transmembrane β-barrel domain. In the case, of long N-, or C-terminal domains mentioned above, we retained only the last or first 12 residues, respectively.

Even in the structures known at atomic resolution, there is not a straightforward way to determine precisely the transmembrane segments, since the lipid bilayer itself is not contained in the crystal structures. This is the case for both α-helical and β-barrel membrane proteins. There are, however a lot of experimentally and theoretically derived sources of evidence, suggesting that the lipid bilayer in gram-negative bacteria, is generally thinner than the bilayer of the inner membrane or those of a typical cell membrane of an eukaryote. Thus, it is believed that the outer membrane possesses an average thickness around 25–30 Å, a fact mainly explainable by its lipid composition, average hydrophobicity and asymmetry [51]. The annotations for the β-strands contained in the PDB entries, are inadequate since there are strands that clearly extend far away from the bilayer. Some approaches have been used in the past, to locate the precise boundaries of the bilayer, but they require visual inspection of the structures and human intervention [23,52]. In order to have objective and reproducible results, we used the annotations for the transmembrane segments deposited in the Protein Data Bank of Transmembrane Proteins (PDB_TM) [53]. The boundaries of the lipid bilayer in PDB_TM have been computed with a geometrical algorithm performing calculations on the 3-dimensional coordinates of the proteins, in a fully automated procedure.

### Prediction methods

The different freely available web-predictors, evaluated in this work, along with the corresponding URLs are listed in Table 6. OM_Topo_predict, is the first Neural Network-based method trained to predict the location of the Cα's with respect to the membrane [14]. Initially, the method was trained on a dataset of seven bacterial porins known at atomic resolution, but later it was retrained in order to include some newly solved (non-porin) structures . B2TMPRED is a Neural Network-based predictor that uses as input evolutionary information derived from profiles generated by PSI-BLAST [15]. The method was trained in a non-redundant dataset of 11 outer membrane proteins, and uses a dynamic programming post processing step to locate the transmembrane strands [54,55]. HMM-B2TMR, is a profile-based HMM method, that was trained for the first time on a non-redundant set of 12 outer membrane proteins [17] and later (current version) on a larger dataset of 15 outer membrane proteins [55]. This method also uses as input profiles derived from PSI-BLAST. It was trained according to a modified version of the Baum-Welch algorithm for HMMs with labeled sequences [56], in order to incorporate the profile as the input instead of the raw sequence, whereas for decoding utilized the posterior decoding method, with an additional post-processing step involving the same dynamic programming algorithm used in B2TMPRED [55]. We should note, that HMM-B2TMR is the only method that currently is available as a commercial demo only, requiring a registration procedure. PRED-TMBB is a HMM-based method developed by our team [19]. Initially, it was trained on a set of 14 outer membrane proteins [19] and later on a training set of 16 proteins [20]. It is the only HMM method trained according to the Conditional Maximum Likelihood (CML) criterion for labeled sequences, and uses as input single sequences. The prediction is performed either by the Viterbi, the N-best algorithm [57] or "a-posteriori" with the aid of a dynamic programming algorithm used to locate both the transmembrane strands and the loops. In this work, we chose to use both N-best and "a-posteriori" decoding, and treat them as different predictors. This was done, since the two alternative decoding algorithms, follow an entirely different philosophy, and in some cases yield different results. BETA-TM, is a simple HMM method trained on 11 non-homologous proteins using the standard Baum-Welch algorithm [58]. It also operates on single sequence mode, and the decoding is performed with the standard Viterbi algorithm. ProfTMB is the last addition to the family of profile-based Hidden Markov Models [21]. It also uses as input evolutionary information, derived from multiple alignments created by PSI-BLAST. It is trained using the modified Baum-Welch algorithm for labeled sequences whereas the decoding is performed using the Viterbi algorithm. Its main difference with HMM-B2TMR, PRED-TMBB, BETA-TM and other previously published, but not publicly available HMM predictors [18], is the fact that it uses different parameters (emission probabilities) for strands having their N-terminal to the periplasmic space, and other for those having their N-terminal to the extracellular space. Furthermore, it uses different states for the modeling of inside loops (periplasmic turns) with different length. TMBETA-NET is a Neural Network based predictor using as input single sequence information [16]. This method uses a set of empirical rules to refine its prediction, in order to eliminate non-plausible predictions for TM-strands (for instance a strand with 3 residues). TBBpred is a predictor combining both NNs and SVMs [22]. The NN-based module also uses evolutionary information, derived from multiple alignments, whereas the SVM-predictor uses various physicochemical parameters. The user may choose one of the methods, or combine them both. The authors of the method have shown, that combining the predictions obtained by NNs and SVMs, improves significantly the prediction accuracy [22]. For the evaluation of the performance and for the Consensus Prediction, we chose to use all three options, in order to investigate which one performs better. Finally, we evaluated the prediction of the transmembrane strands, obtained from a top-scoring general-purpose secondary structure prediction algorithm. This was done, in order to investigate systematic differences in the prediction of the transmembrane β-strands, but also because experimentalists continuously use such algorithms in deciphering assumed topologies for newly discovered β-barrel membrane proteins [59-61]. For this purpose, we have chosen PSI-PRED, a method based on Neural Networks, using multiple alignments derived from PSI-BLAST for the prediction, that has been shown to perform amongst the top-scoring methods for secondary structure prediction [62]. Other, equally successful methods such as PHD [63], perform similarly but they are not considered here.

### Measures of accuracy

For assessing the accuracy of the prediction algorithms several measures were used. For the transmembrane strand predictions we report the well-known *SOV *(measure of the segment's overlap), which is considered to be the most reliable measure for evaluating the performance of secondary structure prediction methods [26]. We also report the total number of correctly predicted topologies (*TOP*), i.e. when both the strands' localization and the loops' orientation have been predicted correctly, and the correctly predicted barrel size (*BS*), i.e the same with the correctly predicted topologies, but allowing for one strand mismatch [20]. As measures of the per residue accuracy, we report here both the total fraction of the correctly predicted residues (*Q_β_*) in a two-state model (transmembrane versus non-transmembrane), and the well known Matthews Correlation Coefficient (*C_β_*) [25].

### Statistical analysis

The measures of accuracy mentioned earlier are the dependent variables that we wish to compare. We treat each prediction on each protein as an observation, and as independent variables we use the type of the submitted sequences (*TYPE*) that could be either the full precursor sequence or the transmembrane barrel domain only, a factor with two categories, and the individual predictive method (*METHOD*), which has 11 categories. Furthermore we tried to group the methods to those based on a Hidden Markov Model and those that were not. This factor (*HMM*) was evaluated later, in order to assess the impact of the type of the prediction method. The formal way to assess the overall statistical significance is to perform a two-way multivariate analysis of variance (MANOVA) [64]. For the evaluation of the statistical significance we evaluated the Wilk's lambda, but the results are not sensitive to this choice since other similar measures (Hotelling-Lawley trace, Roy largest root e.t.c) gave similar results. A statistical significant result, for both the 2 factors (*TYPE*, *METHOD*), will imply that the vector of the measured attributes varies significantly across the levels of these factors. We also included into the models, the interaction term between the two factors (TYPE*METHOD or TYPE*HMM). This was necessary in order to investigate, the potential differences of the dependent variables in the various combinations of the independent variables. For instance, a significant interaction of TYPE with HMM, will indicate that the effect of the input sequence will be different on the two types of methods.

Having obtained a significant result from the MANOVA test, we could use a standard 2-way analysis of variance (ANOVA) for each of the dependent variables, in order to be able to confirm which one of the measured attributes, varies significantly across the two factors. In the ANOVA models, we also included the interaction terms. In all cases, statistically significant results were declared those with a p-value less than 0.05. We report for the ANOVA and MANOVA models, the test statistic and the corresponding p-value, for the fitted models (including the interaction term).

### The consensus prediction method

In order to produce a combined prediction, we have two alternatives: One is to use some kind of ensemble Neural Network, or, alternatively, to summarize the individual predictions using a consensus method. Ensemble Networks show a number of significant advantages over the consensus methods [65,66], but suffer for the limitation that each individual predictor has to be available, every time that a request is made. Since we are dealing with web-based predictors, and we do not have the option to have local copies of each predictor installed, this could be disastrous, thus, the consensus method is the only available and reliable solution.

Suppose we have an amino acid sequence of a protein with length *L*, denoted by:

**x **= *x*_1_, *x*_2_,..., *x*_*L*_,

and for each residue *i *we have the prediction of the *j*_*th *_predictor (*j *= *1, 2, ..., 7*)


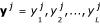


where, 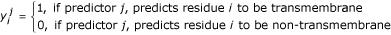


Thus, we can define a per-residue score *S*_*i *_by averaging over the independent contributions of each predictor:


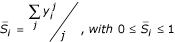


This way, we can obtain a consensus prediction score for the whole sequence,


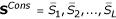


This score is capable of yielding inconsistent predictions, such as a strand with 3 residues for example. For this reason it is then submitted to a dynamic programming algorithm, to locate precisely the transmembrane strands. The algorithm is essentially the same used by [19], with the major difference being the fact that it considers only two states (transmembrane vs. non-transmembrane). It optimizes the predicted topology, according to some predefined parameters, imposed by the observed structures. We also force the algorithm to consider as valid only topologies with an even number of transmembrane strands, as those observed in the crystallographically solved structures. Having determined the number of the transmembrane strands, the final choice of the topology is based on the consideration of the length of the predicted loops. As it has already been mentioned for the 3-dimensional structures, the periplasmic loops have significantly lower length than the extracellular ones, thus by comparing the total length of the two alternative topologies, we decide for the final orientation of the protein.

## Authors' contributions

PGB conceived of the study, performed the collection and analysis of the data and drafted the manuscript, TDL participated in data collection, implemented the consensus algorithm and designed the web interface and SJH supervised and coordinated the whole project. All authors have read and accepted the final manuscript.

## Figures and Tables

**Table 1:** Obtained accuracy of the predictors, in a test set of 20 outer membrane proteins

**METHOD**	**TYPE**	**SEQUENCE**	**Q_β_**	**C_β_**	**SOV**	**Correctly predicted topologies**	**Correctly predicted barrel size**
HMM-B2TMR	HMM	barrel	0.737	0.557	0.836	15	17
		precursor	0.790	0.600	0.813	14	16
ProfTMB	HMM	barrel	0.734	0.537	0.818	14	17
		precursor	0.777	0.575	0.784	12	16
PRED-TMBBpost	HMM	barrel	**0.818**	**0.630**	**0.886**	**14**	**19**
		precursor	**0.842**	**0.637**	**0.852**	**14**	**16**
PRED-TMBBnbest	HMM	barrel	0.818	0.629	0.877	12	17
		precursor	0.849	0.637	0.856	11	13
TBBPred-comb	NN+SVM	barrel	0.702	0.428	0.664	0	0
		precursor	0.701	0.424	0.496	0	0
TBBPred-nn	NN	barrel	0.735	0.466	0.672	0	1
		precursor	0.726	0.432	0.496	0	1
TBBPred-svm	SVM	barrel	0.744	0.458	0.721	1	3
		precursor	0.744	0.426	0.535	0	0
B2TMPRED	NN	barrel	0.723	0.498	0.738	7	9
		precursor	0.709	0.466	0.551	0	0
TMBETA-NET	HMM	barrel	0.697	0.415	0.698	3	8
		precursor	0.663	0.353	0.515	0	4
BETA-TM	NN	barrel	0.690	0.395	0.691	1	2
		precursor	0.663	0.322	0.497	0	1
PSI-PRED	NN	barrel	0.731	0.484	0.690	0	0
		precursor	0.756	0.495	0.569	0	0
HMM-B2TMR, ProfTMB, PRED-TMBBpost, B2TMPRED, TBBPred-nn	CONSENSUS	barrel	**0.819**	**0.641**	**0.924**	**18**	**20**
		precursor	**0.849**	**0.660**	**0.874**	**15**	**18**

For an explanation of the measures of accuracy see the *Materials and Methods *section. Abbreviations: PRED-TMBBpost: PRED-TMBB method with posterior decoding, PRED-TMBBnbest: PRED-TMBB method with NBest decoding, TBBPred-nn: The Neural Network module of TBBPred, TBBPred-svm: The SVM module of TBBPred, TBBPred-comb: TBBPred, combining the Neural Network and SVM modules. The performance of the best individual predictor, and the best available consensus obtained are highlighted with bold.

**Table 2:** Multivariate Analysis of Variance (MANOVA) using as dependent variables the vector of the 5 measures of accuracy.

**A.**	**Wilk's Λ**	**df1**	**df2**	**F**	**p-value**
**overall**	0.1981	105	2029	7.59	<10^-4^
**type**	0.8455	5	414	15.13	<10^-4^
**method**	0.2582	50	1891	13.08	<10^-4^
type*method	0.8541	50	1891	1.33	0.0619

**B.**					

**overall**	0.4511	15	1193	26.58	<10^-4^
**type**	0.8609	5	432	13.96	<10^-4^
**hmm**	0.5441	5	432	72.40	<10^-4^
type*hmm	0.9585	5	432	3.74	0.0025

A. Model that includes as independent variables the individual methods (11 factors), the type of the sequence (barrel/precursor) and their interaction term. B. Model that includes as independent variables the type of the method (HMM/not-HMM), the type of the sequence (barrel/precursor) and their interaction term. We report the Wilk's lambda statistic (Wilk's Λ), the degrees of freedom of the numerator (df1), the degrees of freedom of the denominator (df2), the F statistic (F) and the corresponding p-value (p-value).

**Table 3:** Univariate Analysis of Variance (ANOVA) using each time as dependent variable each one of the 5 measures of accuracy.

	**Q_β_**		**C_β_**		**SOV**		**Correctly predicted topologies**		**Correctly predicted barrel size**	
**A.**	**F**	**p-value**	**F**	**p-value**	**F**	**p-value**	**F**	**p-value**	**F**	**p-value**

**overall**	15.8	<10^-4^	13.55	<10^-4^	13.33	<10^-4^	19.07	<10^-4^	27.34	<10^-4^
**type**	0	0.9444	5.25	0.0224	56.86	<10^-4^	5.51	0.0193	14.97	0.0001
**method**	31.26	<10^-4^	26.97	<10^-4^	20.25	<10^-4^	38.49	<10^-4^	54.14	<10^-4^
type*method	1.93	0.0402	0.96	0.4758	2.05	0.0272	1.01	0.4318	1.77	0.0645

**B.**										

**overall**	27.13	<10^-4^	32.43	<10^-4^	58.18	<10^-4^	72.27	<10^-4^	123.71	<10^-4^
**type**	0.06	0.8144	3.49	0.0625	45.22	<10^-4^	4.33	0.0379	12.28	0.0005
**hmm**	77.59	<10^-4^	91.52	<10^-4^	113.97	<10^-4^	212.4	<10^-4^	358.84	<10^-4^
type*hmm	3.8	0.052	1.79	0.1822	10.83	0.0011	0.01	0.9428	0.1	0.7502

A. Model that includes as independent variables the individual methods (11 factors), the type of the sequence (barrel/precursor) and their interaction term. B. Model that includes as independent variables the type of the method (HMM/not-HMM), the type of the sequence (barrel/precursor) and their interaction term. We report the F statistic (F) of the ANOVA test and the corresponding p-value (p-value).

**Table 4:** Obtained accuracy of the consensus predictions, in the test set of 20 outer membrane proteins

**METHOD**	**TYPE**	**SEQUENCE**	**Q_β_**	**C_β_**	**SOV**	**Correctly predicted topologies**	**Correctly predicted barrel size**
PRED-TMBB, ProfTMB, HMM-B2TMR	CONSENSUS	barrel	0.771	0.596	0.877	17	19
		precursor	0.818	0.628	0.86	15	18
PRED-TMBB, ProfTMB, HMM-B2TMR, B2TMPRED	CONSENSUS	barrel	0.790	0.616	0.896	17	19
		precursor	0.832	0.641	0.865	15	18
PRED-TMBB, ProfTMB, HMM-B2TMR, TBBPred-nn	CONSENSUS	barrel	0.809	0.635	0.917	18	20
		precursor	0.839	0.653	0.867	15	18
PRED-TMBB, ProfTMB, HMM-B2TMR, TBBPred-svm	CONSENSUS	barrel	0.809	0.629	0.906	15	19
		precursor	0.847	0.658	0.882	15	18
PRED-TMBB, ProfTMB, HMM-B2TMR, TBBPred-comb	CONSENSUS	barrel	0.791	0.607	0.894	17	20
		precursor	0.833	0.648	0.859	15	18
PRED-TMBB, ProfTMB, HMM-B2TMR, TBBPred-nn/svm	CONSENSUS	barrel	0.824	0.638	0.92	17	19
		precursor	0.85	0.647	0.871	13	17
PRED-TMBB, ProfTMB, HMM-B2TMR, B2TMPRED, TBBPred-nn/svm	CONSENSUS	barrel	0.825	0.637	0.927	17	18
		precursor	0.854	0.652	0.876	15	17
PRED-TMBB, ProfTMB, HMM-B2TMR, B2TMPRED, TBBPred-nn	CONSENSUS	barrel	**0.819**	**0.641**	**0.924**	**18**	**20**
		precursor	**0.849**	**0.660**	**0.874**	**15**	**18**
PRED-TMBB, ProfTMB, HMM-B2TMR, B2TMPRED, TBBPred-comb	CONSENSUS	barrel	0.807	0.625	0.907	17	19
		precursor	0.845	0.658	0.868	15	18
PRED-TMBB, ProfTMB, HMM-B2TMR, B2TMPRED, TBBPred-svm	CONSENSUS	barrel	0.819	0.637	0.910	15	19
		precursor	0.853	0.659	0.880	14	18
PRED-TMBB, ProfTMB, B2TMPRED, TBBPred-svm/nn	CONSENSUS	barrel	0.829	0.642	0.923	17	18
		precursor	0.851	0.648	0.861	15	16
PRED-TMBB, ProfTMB, B2TMPRED, TBBPred-svm, TBBPred-nn, HMM-B2TMR, TMBETA-NET, PSI-PRED, BETA-TM	CONSENSUS	barrel	0.808	0.582	0.851	11	13
		precursor	0.844	0.604	0.841	12	13

We report the consensus of all the available methods, and the ones that were obtained using the 3 top-scoring HMMs combined in various ways with some of the top-scoring NN/SVM methods. The best results are highlighted with bold. For abbreviations see also Table 1.

**Table 5:** The non-redundant data set of 20 β-barrel outer membrane proteins used in this study.

**Protein name**	**Number of β-strands**	**PDB ID**	**Reference**	**Organism**
NspA	8	1P4T	[67]	*Neisseria Meningitidis*
OmpX	8	1QJ8	[68]	*Escherichia coli*
Pagp	8	1MM4	[69]	*Escherichia coli*
OmpA	8	1QJP	[50]	*Escherichia coli*
OmpT	10	1I78	[70]	*Escherichia coli*
OpcA	10	1K24	[71]	*Neisseria Meningitidis*
Nalp	12	1UYN	[41]	*Neisseria Meningitidis*
OmpLA	12	1QD5	[72]	*Escherichia coli*
Porin	16	2POR	[73]	*Rhodobacter capsulatus*
Porin	16	1PRN	[74]	*Rhodopseudomonas blastica*
OmpF	16	2OMF	[75]	*Escherichia coli*
Osmoporin	16	1OSM	[76]	*Klebsiella pneumoniae*
Omp32	16	1E54	[77]	*Comamonas Acidovorans*
Phosphoporin	16	1PHO	[78]	*Escherichia coli*
Sucrose porin	18	1A0S	[79]	*Salmonella typhimurium*
Maltoporin	18	2MPR	[80]	*Salmonella typhimurium*
FhuA	22	2FCP	[46]	*Escherichia coli*
FepA	22	1FEP	[47]	*Escherichia coli*
FecA	22	1KMO	[48]	*Escherichia coli*
BtuB	22	1NQE	[49]	*Escherichia coli*

**Table 6:** The available predictors, used for predicting the transmembrane strands of β-barrel outer membrane proteins.

Method	Reference	Type	TM Strands	TM Strands + Orientation	Discrimination	URL
B2TMPRED	[15]	NN	x	-	-	
HMM-B2TMR (1)	[17]	HMM	x	x	-	(1)
OM_Topo_predict (2)	[14]	NN	x	x	-	(2)
PRED-TMBB	[19, 20]	HMM	x	x	x	
ProfTMB	[21]	HMM	x	x	x	
TBBpred	[22]	NN+SVM	x	-	x	
BETA-TM	[58]	HMM	x	x	-	
TMBETA-NET	[16]	NN	x	-	-	
PSI-PRED	[62]	NN	-	-	-	

We list the name of the predictor, the reference paper, the type of the method (HMM, NN or SVM), whether it predicts the transmembrane strands, the full topology (TM strands+orientation) and if they are capable of discriminating between β-barrel membrane proteins from non-β barrel membrane proteins.

(1) HMM-B2TMR is available as a commercial demo only.

(2) The OM_Topo_predict web server was not operational, at the time when this research was conducted.
